# Gleanings in Science and Medicine

**Published:** 1893-06-17

**Authors:** 


### Gleanings in Science and Medicine.

Herr Kustermann publishes the result of some experiments on the subject of phthisis in prisons. In a prison where, although a system of disinfection was carefully pursued, cases of phthisis continued to occur frequently, the walls of the cells were sponged, and the moisture injected into the abdomen of the guinea pig. The animals did not become tubercular. Herr Kustermann is probably not far wrong in suggesting that the presence of dried sputum in minute quantities was not the chief cause of phthisis among the prisoners, and that confined air, low spirits, and other predisposing causes were mostly to blame; but, after all, his results are merely negative. Dust taken from cells in which prisoners suffering from phthisis were confined has already been found to contain tubercle bacilli, and Herr Kustermann has only shown that the injection of such dust into the guinea pig does not cause that animal to become tuberculous.

The *Educational Times* calls attention to the work of the Committee on Mental and Physical Condition of Children, which has its office at the Parkes Museum, Margaret Street, W. A report on 50,000 children, who have been seen by Dr. Francis Warner, has been compiled, and will shortly be published. Of these it appears that nearly 18,000 exhibited symptoms of either bodily or mental defects; and we are not in the least surprised to hear it. "Educational failures," it is pointed out, "are often due to a want of knowledge of the physical and mental differences among children." But, under our present system of education, all children, at least all the children of the poor, are educated as if there were no differences whatever in their mental or bodily qualifications. In the Education Department, more than in any other, red tape rules supreme. The school inspector is, as a rule, a doctrinaire. The teacher has no option but to do as he is directed; and the system of payment of Government grants by results is, we believe, one fatal to the health and to the future of many a promising child. Any attempt to make an independent examination of the mental and physical condition of the English school-boy and school-girl of to-day ought to be encouraged, but we fear that to convince the Education Department is a task which nothing but a great popular movement will accomplish.

At this time of year, particularly in a dry season like the present, in the Ascot week, with Henley approaching and the garden party season in full swing all over the country, many persons doubtless suffer from the effects of the common delusion that water is purified by freezing. When purchasing "clear ice," or quenching our thirst in the cool seductive champagne or claret cup, it would be as well to recollect that, in point of fact, ice formed by the freezing of impure water retains rather more than thirty-four per cent. of organic, and about twenty per cent. of inorganic, matter. The obvious moral of this is that ice should not be consumed, as it too often is, recklessly and without regard to the source from which it has been obtained.

Another common delusion, which may lead to dangerous results is a very dry summer is that water is made thoroughly fit for drinking by being boiled. The idea is that boiling destroys the life of the animalculæ from wh ch danger is apprehended and that, therefore, a household in which the water used for culinary and drinking purposes has been boiled is safe. But granting, though this is by no means certain in any case, that all living germs have been killed by the boiling, the minute dead bodies still remain in the liquid. These can only be got rid of by thorough filtering. This is the only real safeguard; and it is worthy of consideration whether some apparatus for filtration might not with advantage be fitted up in the main cisterns from which water is drawn for domestic purposes. The movable hand filters, however effective, are, it is to be feared, often of no real benefit owing to the carelessness of servants, who simply neglect to fill them. The use of cisterns, fitted up with filters at the point of exit, and at the same time properly protected from the dangers of pollution from the air of the house, and cleaned at regular periods, would be a great benefit in places where access to a supply of pure natural water is impossible.

